# Acute Oxygen-Sensing *via* Mitochondria-Generated Temperature Transients in Rat Carotid Body Type I Cells

**DOI:** 10.3389/fphys.2022.874039

**Published:** 2022-04-13

**Authors:** Ryan J. Rakoczy, Clay M. Schiebrel, Christopher N. Wyatt

**Affiliations:** Department of Neuroscience, Cell Biology and Physiology, Wright State University, Dayton, OH, United States

**Keywords:** carotid body, mitochondria, type I cell, hypoxia, temperature, oxygen-sensing

## Abstract

The Carotid Bodies (CB) are peripheral chemoreceptors that detect changes in arterial oxygenation and, via afferent inputs to the brainstem, correct the pattern of breathing to restore blood gas homeostasis. Herein, preliminary evidence is presented supporting a novel oxygen-sensing hypothesis which suggests CB Type I cell “hypoxic signaling” may in part be mediated by mitochondria-generated thermal transients in TASK-channel-containing microdomains. Distances were measured between antibody-labeled mitochondria and TASK-potassium channels in primary rat CB Type I cells. Sub-micron distance measurements (TASK-1: 0.33 ± 0.04 µm, n = 47 vs TASK-3: 0.32 ± 0.03 µm, n = 54) provided evidence for CB Type I cell oxygen-sensing microdomains. A temperature-sensitive dye (ERthermAC) indicated that inhibition of mitochondrial activity in isolated cells caused a rapid and reversible inhibition of mitochondrial thermogenesis and thus temperature in these microdomains. Whole-cell perforated-patch current-clamp electrophysiological recordings demonstrated sensitivity of resting membrane potential (Vm) to temperature: lowering bath temperature from 37°C to 24°C induced consistent and reversible depolarizations (Vm at 37°C: -48.4 ± 4.11 mV vs 24°C: -31.0 ± 5.69 mV; n = 5; *p* < 0.01). These data suggest that hypoxic inhibition of mitochondrial thermogenesis may play an important role in oxygen chemotransduction in the CB. A reduction in temperature within cellular microdomains will inhibit plasma membrane ion channels, influence the balance of cellular phosphorylation–dephosphorylation, and may extend the half-life of reactive oxygen species. The characterization of a thermosensory chemotransduction mechanism, that may also be used by other oxygen-sensitive cell types and may impact multiple other chemotransduction mechanisms is critical if we are to fully understand how the CBs, and potentially other oxygen-sensitive cells, respond to hypoxia.

## Introduction

The carotid bodies (CB) are considered the primary peripheral chemoreceptors. They respond to low blood oxygen levels by stimulating the carotid sinus nerve (CSN) which causes downstream modulation of the pattern of breathing and restoration of blood gas homeostasis. The CBs are vital for the initiation of the hypoxic ventilatory response, are underdeveloped in babies who pass from sudden infant death syndrome and are implicated in the pathogenesis of several diseases (Fletcher, 2000; Porzionato et al., 2013; Del Rio et al., 2016).

Carotid body Type I cells respond to hypoxic stimuli by initiating a chemotransductive mechanism that ultimately leads to closure of TASK potassium ion channels, cellular depolarization, voltage-gated calcium influx, and neurotransmitter release onto the CSN (Buckler, 1997; Buckler, Williams, & Honore, 2000; Kim et al., 2009). Within the Type I cell, a large nucleus confines a dense mitochondrial population to a space directly under the plasma membrane: a microenvironment coined, “the oxygen-sensing micro-domain” (Hellström, 1975; Gao et al., 2017). Within this microenvironment, an interplay between mitochondrial products and TASK channels has been proposed by several groups to mediate the hypoxia-sensing chemotransductive pathway. For example, hypoxia, and other mitochondrial inhibitors, have been shown to stall electron chain transport and cause accumulated [ROS]i and decrease available [ATP]i, both of which have been suggested to attenuate TASK channel activity leading to cellular excitation (Williams & Buckler, 2004; Gao et al., 2017; Prabhakar et al., 2018; Rakoczy & Wyatt, 2018). Recently, hypoxic inhibition of mitochondrial activity has also been suggested to decrease temperature within intracellular microdomains (Chrétien et al., 2018). As TASK channel current has also been shown to be temperature sensitive (Maingret et al., 2000), it was hypothesized that hypoxic inhibition of mitochondrial thermogenesis would lead to CB Type I cellular excitation.

These preliminary experiments provide the first measurements of CB Type I cell TASK channel-mitochondria microdomains and explore whether TASK channels reside at a thermally relevant distance from thermogenic and oxygen-sensitive mitochondria (Sotoma et al., 2021). Using a heat-sensitive, fluorescent dye to visualize oxygen-sensitive thermal transients in Type I cells it was possible to record intracellular temperature changes during mitochondrial inhibition. Finally, current-clamp electrophysiology was used to investigate whether reductions in temperature could initiate whole-cell depolarizations. These pilot studies begin to characterize a novel, oxygen-sensitive thermal-transient signaling pathway that may underpin, in part, a chemotransduction mechanism used by CB Type I cells and other oxygen-sensitive cells (e.g., pulmonary arterial smooth muscle cells) to sense low-oxygen levels.

## Materials and Methods

### Carotid Body Dissection and Type I Cell Isolation

All work performed on animals was conducted in accordance and with approval of Wright State University’s Institutional Animal Care and Use Committee. Carotid bodies and then Type I cells were isolated from ninety two rats across nine litters. Carotid bodies were resected from anesthetized (5% isoflurane in oxygen; 1 L/min) neonatal Sprague-Dawley rats (11–23 days old) before the sedated animals were euthanized via decapitation, see Burlon et al. (2009). Carotid bodies were held in ice-cold Dulbecco’s Phosphate Buffered Saline (DPBS; without calcium or magnesium, Gibco) before Type I cells were dissociated using a digestive enzyme solution composed of: 4mg of collagenase type I (220-240 u/mg, Worthington), and 2mg trypsin from bovine pancreas (8000-12,000 u/mg, Sigma) dissolved in 9.65 ml of DPBS (0.65 ml with calcium and magnesium, 9 ml without calcium and magnesium; Gibco). Isolated CB Type I cells were maintained in Ham’s F12 media (supplemented with 10% fetal bovine serum) and plated onto poly-d-lysine-coated (0.1 mg/ml aqueous, Sigma-Aldrich): 22 × 22 mm microscope glass coverslips (Fisherbrand) for immunocytochemistry experiments, 10mm glass FluoroDish coverslips (WPI) for thermal imaging experiments, or 15 mm round no.1 glass coverslips (Warner Instruments) for electrophysiology experiments. Cells were held in an incubator at 37°C, 5% CO_2_ in humidified air until used for experimentation (2–6 h). These methods reliably isolate viable CB Type I cells that retain their oxygen-sensitivity (Burlon et al., 2009).

### Immunocytochemistry

Glass coverslips with CB Type I cells adhered were removed from the incubator and immediately submerged in freezing methanol (−20 °C) for 15 min, then washed three consecutive times (5 minutes each) with a blocking-buffer consisting of 1% bovine serum albumin (Sigma) and 0.3% Triton X-100 (Alfa Aesar A16046) in DPBS with calcium and magnesium (Gibco). Freezing methanol fixation was selected to fix the cells due to its ability to preserve antibody binding sites. However, no fixing method is perfect (Whelan and Bell, 2015) and future studies using alternate fixing methods would confirm or contrast our data. Mitochondria and TASK-1 *or* Mitochondria and TASK-3 potassium channels were immunofluorescently labeled in separate but identical experiments by first incubating cells with primary antibodies (diluted in blocking-buffer): anti-TOMM20 (1:100 dilution, Abcam AB56783); anti-KCNK3 (1:500 dilution, Alomone APC-024); anti-KCNK9 primary antibody (1:500 dilution, Alomone APC-044), respectively, for 14 h at 4°C in a humidified chamber. Coverslips were then washed with blocking buffer four separate times, for 5 minutes each before secondary antibodies (diluted in blocking-buffer) were applied for TOMM20 (1:400, Alexa Fluor 594, Invitrogen A21201; excitation/emission: 594 nm/617 nm), and TASK-1 or TASK-3 (1:500, Alexa Fluor 488, Invitrogen A21441; excitation/emission: 493 nm/519 nm) and left at room temperature in darkness for 2 hours. Secondary antibodies were washed from coverslips using phosphate buffered saline with calcium and magnesium (Gibco), five separate times for 5 minutes each. In separate control experiments, primary antibodies were omitted, and only secondary antibodies exposed to cells to ensure no cross-reactivity or off targeted binding, of which none was observed (i.e., no signal present when imaged; data not shown). Micro slides (25 × 75 × 1 mm thick, VWR 48312) were labeled and a drop (∼50 µL) of DAPI-containing Vectashield Antifade Mounting Media (Vector Laboratories) was added before coverslips were mounted and then sealed with clear nail polish. Slides were then protected from light and stored at 4°C until imaged (1–7 days post-staining).

### Confocal Distance Measurements

Slides with immunofluorescent-labeled TASK potassium channels (TASK-1 or TASK-3) and mitochondria (TOMM20) were placed on a confocal microscope stage (Olympus FV1000 Confocal LSM). Cells were visualized using a ×60 objective (Nikon), and TASK-1 or TASK-3 staining via Alexa Fluor 488 was visualized with a 488 nm laser line (15% laser power, HV: 700), and mitochondrial staining (TOMM20) via Alexa Fluor 594 was visualized with a 561 nm laser line (15% laser power, HV: 700). Z-stack (50nm steps) images were acquired sequentially for each channel, Kalman filter was activated (set to average two by line; reduces noise), aspect ratio of 1024 × 1024 pixels, 7.5x zoom, pixel size X/Y: 47 nm^2^, and 4.0 µs/pixel dwell time. Raw images were saved in a hyperstack (Olympus.oib format) with microscope parameters and imaging data included in an Olympus metafile. Image deconvolution was accomplished using Huygens Essential Software (SVI, Hilversum, Netherlands v4.2), separately for each channel per z-stack image, using the Classic Maximum Likelihood Estimation algorithm (maximum iterations: 30). Deconvolved images were next imported into Imaris computer software (Oxford Instruments Imaris v7.7.0) and three-dimensional, volumetric rendering was accomplished via software generation of maximum intensity projections. Voxels with fluorescent-signal meeting software criterion for illumination were rendered as voluminous red or green objects (representative of mitochondria or TASK-channels, respectively) and visualized in a 3D-modeled arena topographically representative of the imaged CB Type I cells. Three-dimensional rendering and visualization of cells allowed measurements to be acquired of distances between mitochondria and TASK-1 or TASK-3 channels (Firth et al., 2009). Imaris software’s “measurement tool” was used to generate measurements, only in the “X-Y” plane, of distances between the surface of a TASK-1 or TASK-3 channel signal (i.e., Alexa Fluor 488) and the surface of the nearest mitochondria signal (i.e., Alexa Fluor 594). Data were recorded, averaged by group (i.e., mitochondria distance to TASK-1 vs TASK-3), and an unpaired student’s t-test used to statistically test if measured distances between mitochondria and respective TASK-channels were significantly different from one another. Data were analyzed and figures generated in software program Prism (Graphpad Prism v9.1.2). Data are presented as the averaged distance measured between CB Type I cell mitochondria and TASK-1 or TASK-3 ion channels plus or minus the standard error of the mean. Statistical significance was denoted if a *p*-value of <0.05 (n.s.: not significant, *p* < 0.05: *, *p* < 0.01: **, *p* < 0.001: ***, *p* < 0.0001: ****, for comparisons in all figures).

### Thermal Imaging

FluoroDish coverslips (FD3510 WPI) with CB Type I cells adhered were removed from the incubator and cells loaded with 250nM ERthermAC (EMD Millipore SCT057). ERthermAC is a commercially available, membrane-permeable and endoplasmic reticulum-targeted, BODIPY-based dye; fluorescence is inversely proportional to change in temperature whereby a decrease in temperature is visualized as a reversible increase in ERthermAC relative signal fluorescence (Kriszt et al., 2017). After loading with ERthermAC, cells were washed twice with 37°C HEPES-buffered extracellular solution (see electrophysiology methods for components), for 10 minutes each. For control experiments, loaded cells were next fixed with 4% paraformaldehyde and washed twice thereafter with 37°C extracellular-solution. Coverslips were then viewed using an Olympus FV1000 microscope. Gravity-fed extracellular solution was perfused (5 ml/min) over the coverslip for the entire length of the experiment with temperature maintained as it was passed through a thermistor-feedback controlled inline heater (Warner Instruments TC-344), set to maintain the solution in the dish at 37°C or 22°C, depending upon the experiment. Images of cells loaded with ERthermAC were acquired over time (every 5 s) with a water-immersion ×25 objective lens (Nikon XLPlan N). Cells were allowed 10 min to equilibrate to the bath (volume 1.0 ml) and baseline measurements were made before rapidly switching to a “stimulus” perfusate (i.e., identical solution with 2mM cyanide added or bubbled with 100% nitrogen and 0.2mM sodium dithionite for the anoxic solution). Regions of interest (ROI) were drawn around whole CB Type I cells (Figure 3) and pixel intensities were spatially averaged post-hoc within ImageJ (NIH). Results were interpreted as group average relative fluorescent signal intensity units (RFU) during time of interest and from baseline fluorescent intensity measurements (averaged in 30-s bins). Paired Student’s t-tests tested for significant differences between the relative fluorescent signal averaged values from peak responses at the time of interest and during baseline recordings, within each cell recording. Significance was determined with a *p*-value < 0.05.

### Electrophysiology

The amphotericin-B (240 μg/ml; Sigma) mediated perforated-patch clamp technique was performed using glass electrodes (resistance: 6.5–9.5 MΩ) back-filled with intracellular solution composed of (in mM): 55 K_2_SO_4_, one EGTA, 30 KCl, 20 HEPES, five MgCl_2_, 10 glucose, adjusted to pH 7.2 with KOH. Coverslips with isolated CB Type I cells adhered were loaded onto a perfusion chamber, mounted over an inverted microscope (Nikon TE2000-U), and gravity-fed, HEPES-buffered solution perfused (5 ml/min) for the entire experiment. Recording chamber volume was 0.2ml. Solution temperature was maintained as it passed through a thermistor-feedback controlled inline heater (Warner Instruments TC-344). HEPES-buffered solutions contained the following (in mM): 140 NaCl, 4.5 KCl, 2.5 CaCl_2_, one MgCl_2_, 11 glucose, 10 HEPES, and were adjusted to pH 7.4 with NaOH at respective temperature. Resting membrane potentials were recorded in current-clamp mode (“I = 0”; gap-free at 2 KHz) in 37°C HEPES-buffered solution before rapidly switching to a 24°C HEPES-buffered solution. All data were analyzed off-line using Clampfit 10 (Molecular Devices). Data were filtered at 1KHz and analyzed in 30-s binned averages at baseline and peak response. Paired Student’s t-tests determined if average resting membrane potentials recorded at 24°C were different from those recorded at 37°C for the same cell. Data are reported as group averages plus or minus the standard error of the mean. Statistical significance was denoted if a *p*-value of <0.05 was observed.

## Results

### TASK-To-Mitochondria Distance Measurements

**FIGURE 1:**
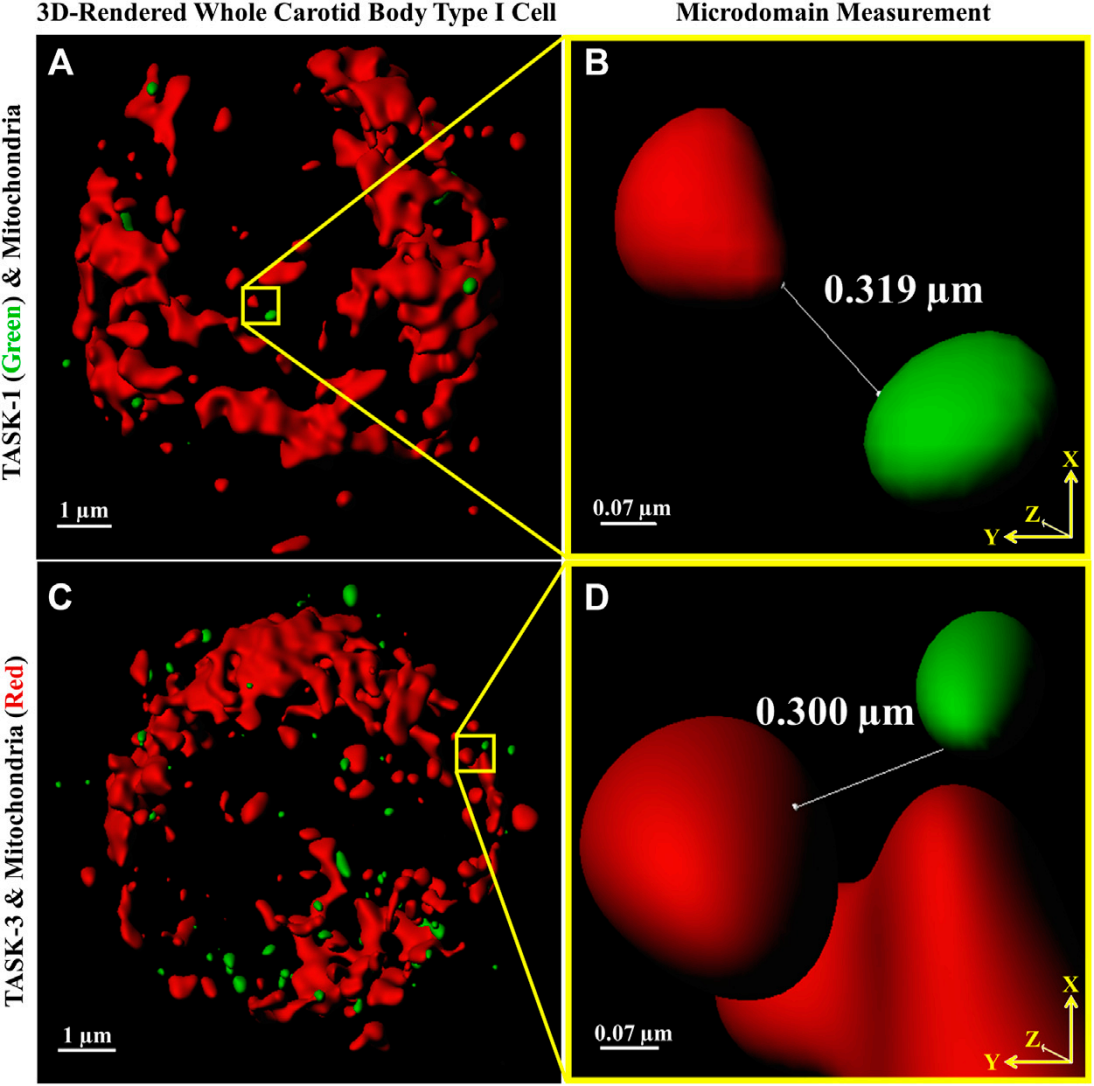
Distances between Carotid Body Type I cell **(A,C)** fluorescently antibody-labeled mitochondria (red) and TASK-1 **(A & B);** green and TASK-3 **(C & D);** green potassium ion channels were measured providing first quantitative evidence of microdomains. Software-provided tools (Imaris) defined line end-points (shown in **B**,**D**) as an intersection of an *x*-*y* axis oriented line with MIP-rendered voxel surface boundaries. Images A and C are 3D reconstructions of whole Type I cells, the nuclei are not stained.

**FIGURE 2:**
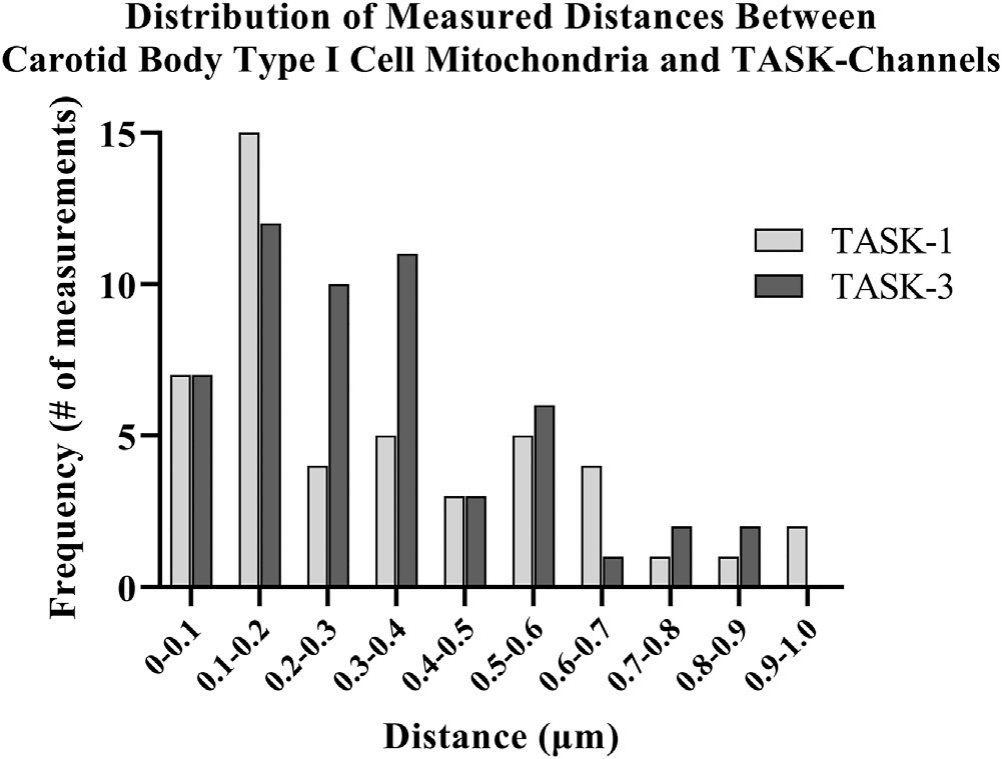
Distances between antibody-labeled (TOMM20) mitochondria and TASK-potassium channels in rat Carotid Body Type I cells were measured using confocal microscopy. Distribution of software-measured distances showed approximately 72% of TASK-1 and approximately 79% of TASK-3 total measurements were ≤0.5 µm.

**FIGURE 3:**
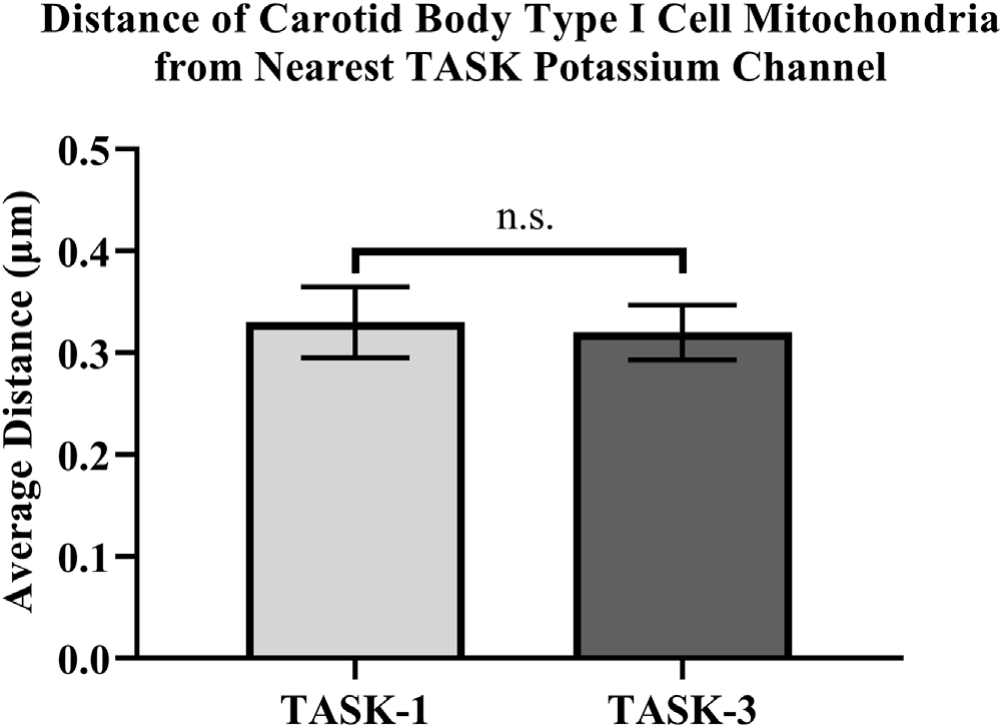
Distances between antibody-labeled (TOMM20) mitochondria and TASK-potassium channels in rat Carotid Body Type I cells were measured using confocal microscopy. Microdomains were found between mitochondria and TASK-1: 0.33 ± 0.04 µm (n = 47) and TASK-3 potassium channels: 0.32 ± 0.03 µm (n = 54).

Distances between fluorescently labeled mitochondria (TOMM20) and TASK-1 (Figure 1 A & B) or TASK-3 channels (Figure 1 C & D) were measured. It was of interest to determine if CB Type I cell oxygen-sensing microdomains formed whereby mitochondria closely associate with TASK-potassium ion channels forming chemotransductive-signaling arenas (Gao et al., 2017; Rakoczy & Wyatt, 2018). TASK-1 and -3-channels were targeted, as they are the major “oxygen-sensitive” current-carriers in rodent CB Type I cells (Kim et al., 2009; Turner & Buckler, 2013). Details of CB Type I cells microdomains, defined as a region whereby a TASK-Channel (i.e., TASK-1 or -3 signal) was within 1 μm of mitochondria (i.e., TOMM20 signal), are provided in Figure 2. Distribution of data in Figure 2 show ∼72% of TASK-1 and ∼79% of TASK-3 measured distances from mitochondria were ≤0.5µm. As a TASK-1/3 heteromultimer-specific antibody is not commercially available, distances between mitochondria and TASK-1 and TASK-3 channels were made separately but in identical experiments. Average distances found between mitochondria and TASK-1: 0.33 ± 0.04µm (n = 47; from 16 cells over six coverslips) and TASK-3 potassium channels: 0.32 ± 0.03 µm (n = 54, from 15 cells over five coverslips), shown in Figure 3, were not significantly different from one another, and suggest both CB Type I cells’ TASK-1 and TASK-3 monomeric, potentially as well as TASK-1/3 heteromultimer potassium ion channels reside in microdomains.

Rapid intracellular thermal fluxes have been demonstrated to occur, in living cells, at up to 3-fold the distances, compared those measured here (i.e., 1 μm: Sotoma et al., 2021). As CB Type I cells contain a dense population of mitochondria, and these organelles are known to produce heat as a byproduct of respiratory chain activity (i.e., mitochondrial thermogenesis; Chrétien et al., 2018), it was of interest that rodent CB Type I cells TASK-Channels resided within distances from a heat-source wherein rapid changes in intracellular temperature have been shown to occur (Sotoma et al., 2021). Therefore “heat flux for short-distance thermal signaling” in CB Type I cells may be possible whereby temperature-sensitive TASK-Channel activity is influenced by oxygen-sensitive mitochondrial thermogenesis (Maingret et al., 2000; Chrétien et al., 2018; Sotoma et al., 2021).

### Thermal Imaging

**FIGURE 4:**
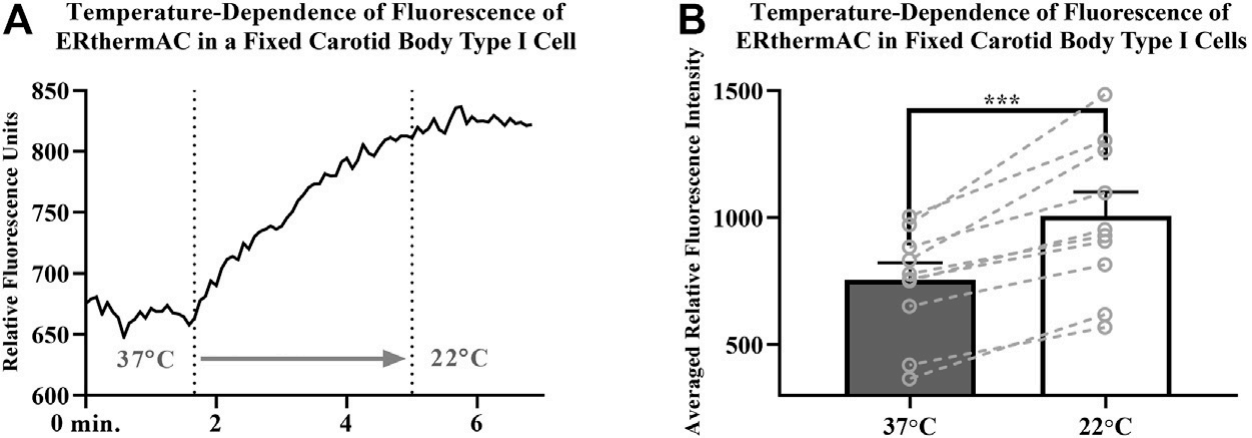
**(A)** Example trace demonstrating temperature-dependence of ERthermAC (250 nM) loaded in an isolated and paraformaldehyde-fixed (4%), Carotid Body Type I cell; relative signal intensity increases as the extracellular solution was switched (first dotted line) from a 37°C HEPES-buffered solution to a 22°C HEPES-buffered solution (second dotted line) **(B) Temperature-**dependence of ERthermAC (250 nM) loaded in paraformaldehyde fixed, primary Carotid Body Type I cells; relative signal intensity was averaged for each condition (37°C vs 22°C; 30 s bins) and fluorescent signal intensity found to significantly increase in fixed cells as they were passively cooled from 37°C to 22°C (*p* < 0.001; n = 10; 755.1 ± 67.3 vs 1007.8 ± 93.7 RFU).

**FIGURE 5:**
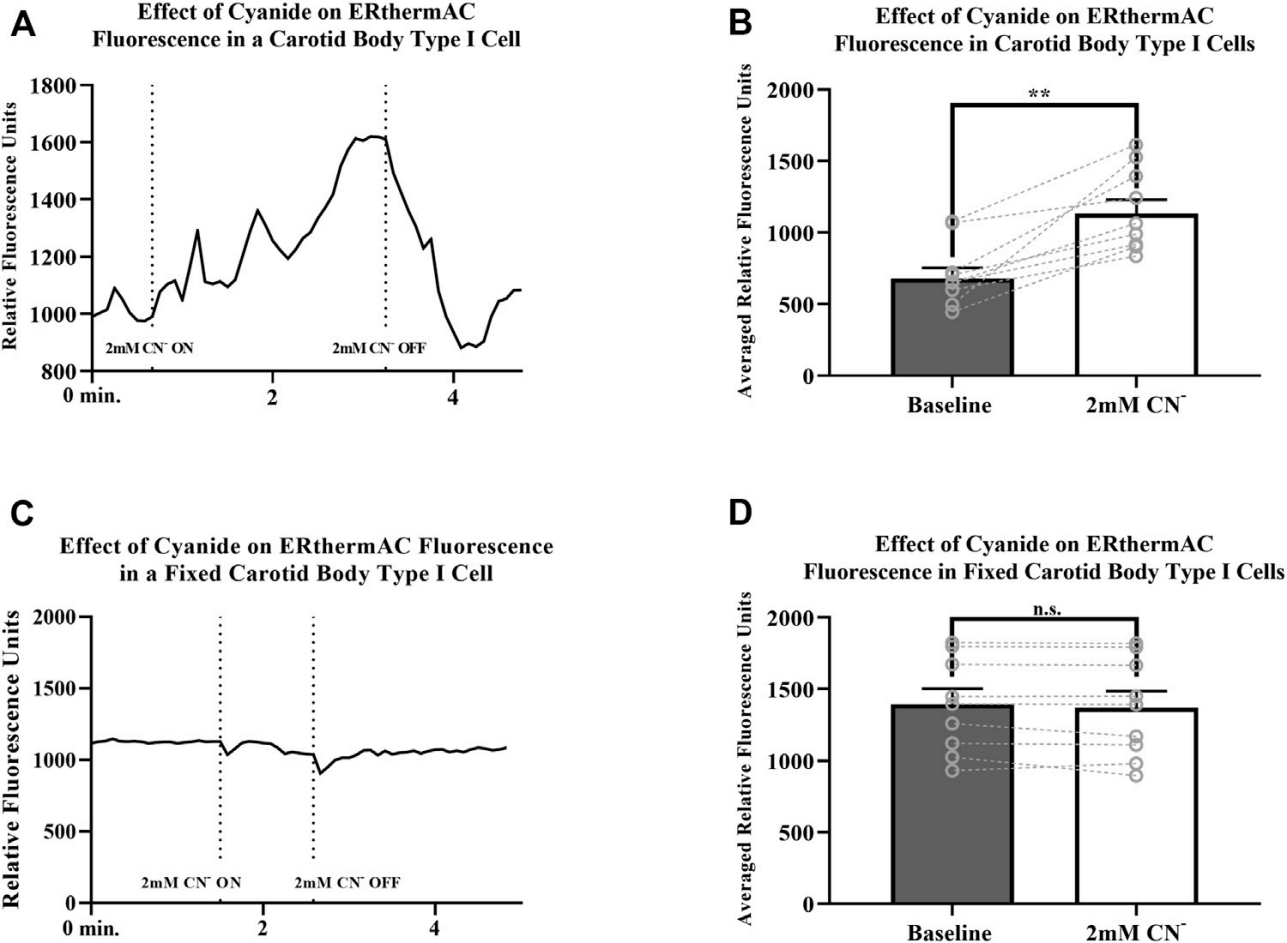
**(A)** Example trace measuring ERthermAC (250 nM) fluorescence from a live, isolated Carotid Body Type I cell exposed to 2mM cyanide (between dotted lines). Relative fluorescent intensity is reversible and increases suggesting a decrease in whole-cell temperature as a result of mitochondrial inhibition. Bath temperature remained constant at 37°C for the duration of the experiment **(B)** Averaged ERthermAC (250 nM) fluorescence signals from live, isolated Carotid Body Type I cells exposed to 2 mM cyanide. Relative fluorescent intensity increases suggesting a decrease in whole-cell temperature as a result of mitochondrial inhibition (*p* < 0.01; n = 9; 679.3 ± 74.3 vs 1131.9 ± 96.6 RFU) **(C)** Example trace measuring ERthermAC (250nM) fluorescence in a fixed, isolated Carotid Body Type I cell exposed to 2 mM cyanide (between dotted lines). No significant change in ERthermAC relative fluorescent intensity is noted when fixed cells were exposed to cyanide containing HEPES-buffered solution in control experiments **(D)** Control experiments showed no significant change in averaged ERthermAC (250 nM) signal fluorescence from baseline responses when fixed Carotid Body Type I cells were exposed to 2 mM cyanide (n.s.; 1391.9 ± 110.2 vs 1370.1 ± 115.5 RFU.; n = 9).

**FIGURE 6:**
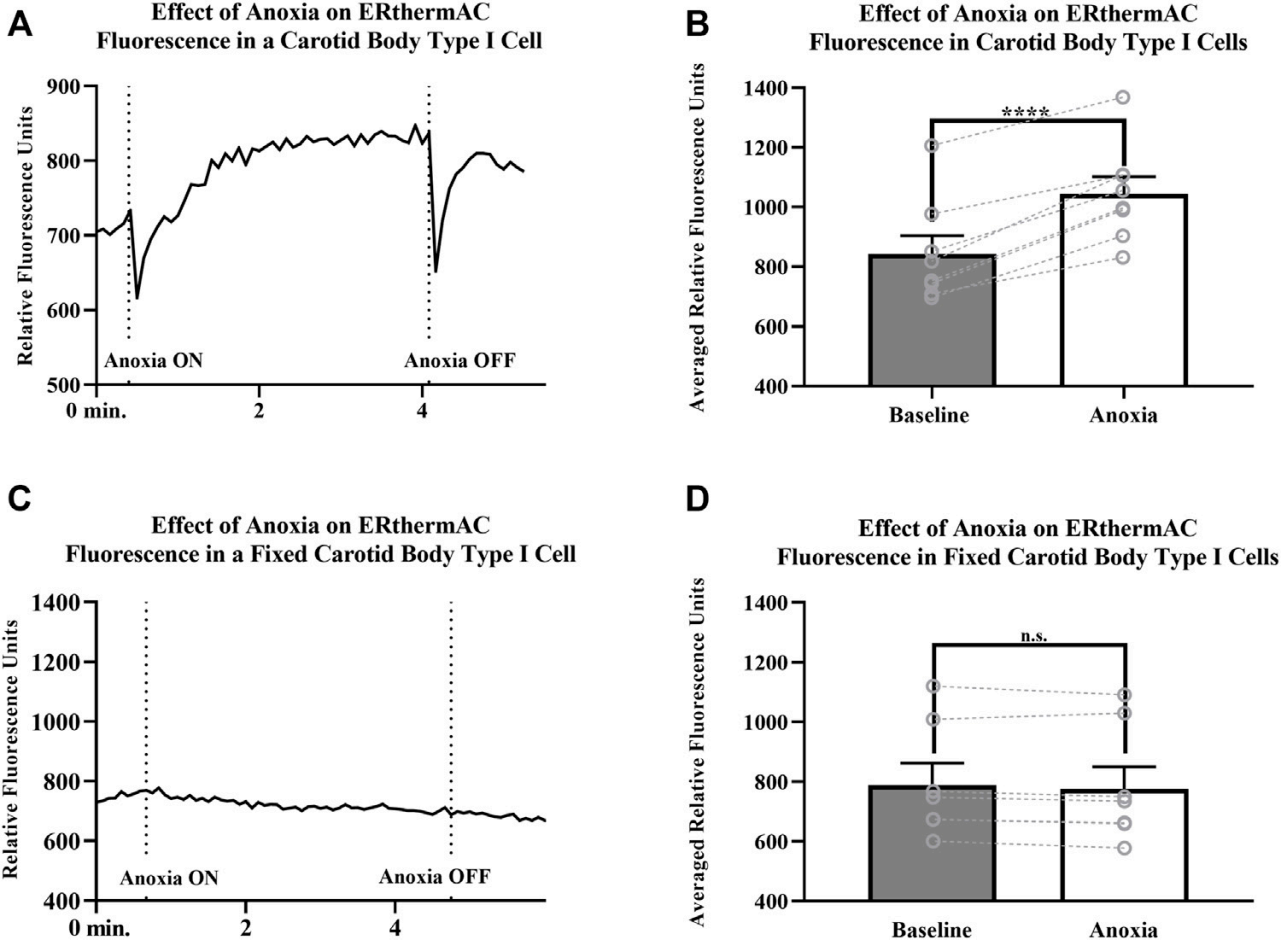
**(A)**ERthermAC (250 nM) fluorescence signals from a live, isolated Carotid Body Type I cell exposed to anoxia. Relative fluorescent intensity increases suggesting a decrease in whole-cell temperature as a result of hypoxic exposure. Bath temperature remained constant at 37°C for the entire length of the experiment **(B)** Averaged ERthermAC (250 nM) fluorescence signals from live, isolated Carotid Body Type I cells exposed to anoxia. Relative fluorescent intensity increases suggesting a decrease in whole-cell temperature as a result of anoxic exposure (*p* < 0.0001, n = 8; 843.3 ± 61.1 vs 1044.8 ± 57.3 RFU) **(C)** Example trace showing ERthermAC (250 nM) fluorescence in a fixed, isolated Carotid Body Type I cell exposed to an anoxic extracellular HEPES-buffered solution. Anoxia did not cause a significant effect on ERthermAC signal fluorescence in fixed Carotid Body Type I cells **(D)** Control experiments showed no significant change in averaged ERthermAC (250 nM) signal fluorescence from baseline responses when paraformaldehyde-fixed Carotid Body Type I cells were exposed to anoxic HEPES-buffered solution (n.s.; 789.1 ± 72.8 vs 776.3 ± 74.4 RFU; n = 7).

Intracellular temperature of isolated CB Type I cells was visualized using the heat-sensitive dye, ERthermAC. The first set of experiments utilized paraformaldehyde-fixed, and previously dye-loaded, CB Type I cells to test the temperature-dependence of the dye in non-living cells. Cells were imaged while the bath temperature was reduced from 37°C to 22°C (example trace shown in Figure 4A). Relative signal intensity was averaged for each condition (37°C vs 22°C; 30 s bins) and fluorescent signal intensity found to significantly increase in fixed cells as they were passively cooled from 37°C to 22°C (*p* < 0.001; n = 10; 755.1 ± 67.3 vs 1007.8 ± 93.7 relative fluorescence intensity units; Figure 4B). ERthermAC signal intensity (250nM) was shown to be inversely related to temperature: as the bath temperature was decreased, signal intensity was found to significantly increase. Next, it was of interest to test whether whole-cell temperature changes occur as a result of inhibition of mitochondrial activity (i.e., electron chain transport). Baseline fluorescent measurements were made before cells were exposed to 2mM cyanide while bath temperature was held constant at 37°C for the entire length of the experiment (example trace shown in Figure 5A). Cyanide was found to significantly increase ERthermAC fluorescence, suggesting a decrease in intercellular temperatures (*p* < 0.01; n = 9; 679.3 ± 74.3 vs 1131.9 ± 96.6 RFU; Figure 5B). Control experiments exposed ERthermAC-loaded, paraformaldehyde-fixed CB Type I cells to 2 mM cyanide (trace shown in Figure 5C) and showed no significant effect of the compound on signal fluorescence (n.s.; 1391.9 ± 110.2 vs 1370.1 ± 115.5 RFU.; n = 9; Figure 5D). As CB Type I cells depolarize in response to low oxygen levels, we next decided to test whether anoxia was capable of decreasing whole-cell temperatures. Baseline measurements were made again in ERthermAC-loaded, isolated CB Type I cells with bath temperature held constant at 37 °C for the entire experiment, before rapidly switching to a hypoxic HEPES-buffered solution (N_2_ bubbled and 0.2mM sodium dithionite added; trace shown in Figure 6A). Anoxia was shown to significantly increase ERthermAC signal intensity, again suggesting a decrease in intercellular temperatures (*p* < 0.0001, n = 8; 843.3 ± 61.1 vs 1044.8 ± 57.3 RFU; Figure 6B). Control experiments exposed ERthermAC-loaded, paraformaldehyde-fixed CB Type I cells to the anoxic solution (trace shown in Figure 6C) and showed no significant effect of anoxia on signal fluorescence (n.s.; 789.1 ± 72.8 vs 776.3 ± 74.4 RFU; n = 7; Figure 6D). Together, results suggested that inhibition of CB Type I cell mitochondrial activity was capable of significantly reducing whole-cell temperatures. Furthermore, as mitochondria in Type I cells are found near temperature sensitive TASK potassium channels (see Figures 1, 2 and Maingret et al., 2000), oxygen-dependent temperature reductions in CB Type I cell microdomains may play a role in chemotransduction in these cells, and potentially others (Firth et al., 2009).

### Electrophysiology

**FIGURE 7:**
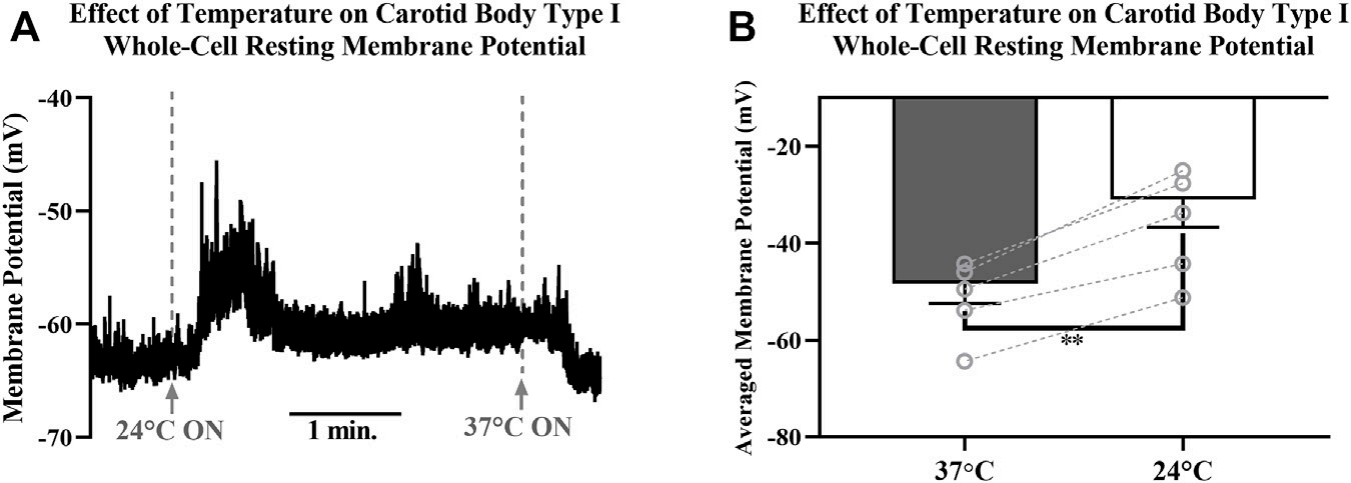
**(A)** Example trace showing the effect decreasing temperature of extracellular HEPES-buffered solution has on resting membrane potential of a Carotid Body Type I cell measured via perforated-patch whole-cell current-clamp recordings. Solutions were initially 37°C, were switched to a 24°C solution at the first dotted line and then returned to 37°C at the second dotted line. Reversible depolarizations were consistently observed when solution temperature was decreased from 37°C to 24°**(B)** Decreasing temperature of extracellular HEPES-buffered solution from 37°C to 24°C caused significant membrane depolarization in isolated Carotid Body Type I cells (-48.4 ± 4.1 mV vs. −31.0 ± 5.7 mV; n = 5; *p* < 0.01) measured in perforated-patch whole-cell current-clamp (I = 0 mode) recordings.

Resting membrane potentials of isolated CB Type I cells were recorded via perforated-patch whole-cell current-clamp (I = 0 mode) recordings. Electrophysiological recordings demonstrated sensitivity of resting membrane potentials to temperature (example trace shown in Figure 7A): lowering the temperature of the extracellular solution from 37° to 24°C induced consistent and reversible cellular depolarizations. Averaged resting membrane potentials measured of isolated CB Type I cells at 37°C were significantly different from depolarized potentials measured when bath temperature was lowered to 24°C (-48.4 ± 4.1 mV vs V_m_ 24°C: -31.0 ± 5.7 mV; n = 5; *p* < 0.01; Figure 7B) this represents an average depolarization of 17.4 ± 2.2 mV.

## Discussion

Results from these preliminary studies provide evidence supporting a novel oxygen-sensing hypothesis suggesting rat CB Type I cell hypoxic signaling may in part be mediated by mitochondria-generated thermal transients in TASK-channel-containing micro-domains. Submicron distance measurements between TASK channels and mitochondria confirm the existence of oxygen-sensing micro-domains and are conducive for rapid diffusion of mitochondrial products (i.e., ROS, ATP) and propagation of heat (Varas, Wyatt, & Buckler, 2007; Gao et al., 2017; Sotoma et al., 2021). Thermal imaging experiments demonstrated mitochondrial thermogenesis is oxygen-dependent and mitochondrial inhibition was observed to significantly decrease intracellular temperatures in isolated cells. Perforated patch-clamp electrophysiological recordings of whole-cell resting membrane potentials demonstrated lowering bath temperature to induce consistent and reversible depolarizations. These initial studies demonstrate that anoxia may cause a change in temperature in cellular microdomains that lead to depolarization of Type I cells. However, much more work needs to be done. Type I cells should be loaded with temperature sensitive dye and then patched. Simultaneous recordings of dye, membrane potential and bath oxygen should be made and cells should be exposed to hypoxia. Following these live cell recordings cells should be fixed and the dye calibrated for temperature. Only by doing this could it be confirmed that hypoxia is changing cell temperature and that temperature change is coincident with depolarization at a specific temperature or range of temperatures.

Future experiments are also necessary to confirm that the major oxygen-sensitive-current-carrying TASK-1/3 heteromultimers reside in micro-domains, as only monomer isoforms were able to be studied. Discovering whether other channels reside in micro-domains would warrant investigation into their temperature sensitivity as well. For example, calcium influx through voltage-gated channels has been shown to be temperature-dependent but the channels have yet to be localized to micro-domains (Allen, 1996; Iftinca et al., 2006). Previous studies have mostly suggested a lack of participation by temperature sensitive transient receptor potential (TRP) channels in Type I cell oxygen-sensing chemotransduction (Roy et al., 2012; Pokorski et al., 2014; Iturriaga et al., 2021). However, the non-selective TRP-channel inhibitor, 2-APB, has been noted to reduce calcium-influx during hypoxia (Kim et al., 2015). Though, as it also inhibits IP3-receptor activation, this is an effect that cannot be entirely ascribed to TRP-channel inhibition (Splettstoesser, Florea, & Büsselberg, 2007).

More recent findings have provided some evidence that TRPM7 may be present in the mouse carotid body (Shin et al., 2019). Importantly, the depolarization of rat Type I cells when exposed to lower temperatures is contrary to what would be predicted if TRPM7 channels were active at the resting membrane potential. Cooling active TRPM7 channels is likely to cause a hyperpolarization and this was not observed (see Figure 7). If TRPM8 (McKemy et al., 2002) were discovered to be present in rat Type I cells then a compelling argument for their involvement in thermal hypoxic-chemotransduction might be made. To date the presence of TRPM8 in rat Type I cells has not been demonstrated.

The data showing reduced temperatures caused Type I cellular depolarization concur with results discussed by Eyzaguirre & Fidone (1980). Eyzaguirre and Fidone noted that cooling caused depolarization presynaptically in Type I cells but paradoxically reduced CSN firing in intact carotid body preparations. The current-clamp data (Figures 7A,B) support these findings and data in Figures 1, 2 provide a potential mechanism by which hypoxia may influence temperature in microdomains thereby leading to depolarization. It is perhaps unsurprising that cooling the whole carotid body reduces firing of the CSN and this is likely via a direct effect on the nerve.

Micro-domains are not unique to CB Type I cells. They have been identified in other oxygen-sensitive cells, such as pulmonary arterial smooth muscle cells (PASMC), whereby mitochondria colocalize with voltage-gated potassium channels (Kv) and influence their activity (Firth et al., 2009). Evidence of the absence of micro-domains in key non-oxygen-sensitive cells, such as guinea pig CB Type I cells, might further suggest that a spatial coupling of oxygen-sensitive mitochondria to effector ion channels (i.e., Kv in PASMC or TASK in CB) underpins cellular oxygen-sensitivity (Blake & Bachero, 1985). Equally it would be intriguing to see if the distance between mitochondria and potassium channels increases as oxygen-sensitive neonatal adrenal chromaffin cells mature into non-oxygen-sensing mature cells. Furthermore, characterizing micro-domains in oxygen-sensitive cells may identify a conserved cytostructure that serves to functionally couple mitochondria to potassium channels (i.e., sensors to effectors), and thus provide a chemotransductive arena used for hypoxic signaling.

These preliminary results provide the first measurements of the hypothesized CB oxygen-sensing micro-domain (Varas, Wyatt, & Buckler, 2007; Gao et al., 2017). As many of the CB oxygen-sensing theories mechanistically hinge upon the proximity of mitochondria to ion channels, direct evidence was needed. The concept that reduced oxygen stimulates CB Type I cells by reducing temperature in microdomains is novel and warrants further, more detailed investigation in a range of acutely oxygen-sensitive tissues.

## Data Availability

The raw data supporting the conclusions of this article will be made available by the authors, without undue reservation.
